# Assessment of quality of life in patients with post kalaazar dermal leishmaniasis

**DOI:** 10.1186/s12955-017-0720-y

**Published:** 2017-07-24

**Authors:** Biplab Pal, Krishna Murti, Niyamat Ali Siddiqui, Pradeep Das, Chandra Shekhar Lal, Rajendra Babu, Manoj Kumar Rastogi, Krishna Pandey

**Affiliations:** 1 Department of Pharmacy Practice, National Institute of Pharmaceutical Education and Research, Hajipur, Bihar India; 2 Department of Biostatistics, Rajendra Memorial Research Institute of Medical Sciences (Indian Council of Medical Research), Agamkuan, Patna, Bihar India; 3 Department of Molecular Biology, Rajendra Memorial Research Institute of Medical Sciences (Indian Council of Medical Research), Agamkuan, Patna, Bihar India; 4 Department of Biochemistry, Rajendra Memorial Research Institute of Medical Sciences (Indian Council of Medical Research), Agamkuan, Patna, Bihar India; 5 Department of Clinical Medicine, Rajendra Memorial Research Institute of Medical Sciences (Indian Council of Medical Research), Agamkuan, Patna, Bihar 800007 India

**Keywords:** Quality of life, Pkdl, India, Dlqi, SF36

## Abstract

**Background:**

Post kala-azar dermal leishmaniasis (PKDL) is a dermatological disorder caused by protozoal parasite *Leishmania donovani*. PKDL cases are thought to be a reservoir of parasites and may increase cases of visceral leishmaniasis. The disease is not life threatening but cosmetic disfigurement associated with it may impair the patients’ quality of life. This study aimed to assess the health related quality of life in patients with post kalaazar dermal leishmanasis for the first time.

**Methods:**

A total of 92 PKDL cases and 96 healthy participants filled out the questionnaires. The Dermatology Life Quality Index (DLQI) and SF 36 questionnaire were used to assess the quality of life. Data on socio-demographic and clinical features were also collected. The collected data were analyzed by using SPSS software (version 16), Student’s t-test, analysis of variance (ANOVA) was applied for comparison of means.

**Results:**

PKDL patients experienced very large impact on their quality of life. The mean score of DLQI was 11.41. Highest impact was found in symptoms and feelings and lowest impact was observed for personal relationship domain. Patients below 20 years age group found to have lower quality of life. There was a significant difference in mean DLQI scores with regard to age and severity of lesions (*P* < 0.05). No significant difference was observed with respect to gender, duration and location of lesions (*p* > 0.05).

**Conclusion:**

PKDL significantly impaired the patient’s quality of life. Further studies to assess the impact of treatment on quality of life in these patients are recommended.

## Background

Leishmaniasis is a vector-borne disease caused by protozoa that belongs to the genus Leishmania. This is one of the most neglected diseases. Its global prevalence is 12 million with 350 million people at risk [1, 2]. The clinical manifestations of leishmaniasis range from benign cutaneous leishmaniasis (CL) to visceral leishmaniasis [3, 4]. Visceral leishmaniasis (VL)/ Kala-azar is one of the World’s most neglected and poverty-related diseases, affecting the poorest people in developing countries associated with malnutrition, weakness of the immune system, displacement, poor housing, illiteracy, gender discrimination and lack of resources [5].

More than 90% of global VL occurs in India, Sudan, Bangladesh, Nepal, Ethiopia and Brazil. In India Visceral leishmaniasis is endemic in North-eastern states like Bihar, Jharkhand, West Bengal, Assam and Uttar Pradesh; sporadic cases are also reported from other states. Post kala-azar dermal leishmaniasis (PKDL) is a dermatological complication occurring among the treated visceral leishmaniasis (VL) patients and also among those without a history of VL. The disease is characterized by hypo-pigmented macule, papule and nodule or a combination of these lesions. In India, PKDL usually occurs at an interval of 2-3 years in 5- 10% of cases after successful treatment of VL [6]. Prevalence of confirmed PKDL cases in Bihar, India is found to be 4.4/10,000 and 7.8/10,000 including probable cases [7].

PKDL is non-fatal and it does not interfere with routine activities of affected individuals, but social stigma associated with the disease makes the life difficult. Patient with visible rashes may suffer from physical discomfort, embarrassment, social isolation and may go into hiding. Thereby, day to day relationship with friends and close relatives may be hampered, which can lead to emotional distress. For girls at the marriageable age cosmetic disfigurement or eroded beauty may decrease the likelihood of getting married [8]. Social stigma is thought to be more when the lesions are present in the uncovered portion of the body [9]. A healthy skin is not only required for mental and physical well being, but simultaneous enhancement of sexual attractiveness and self confidence of an individual. The Chronic dermatological disorder may profoundly affect the physical, emotional and social well being of an individual. Patients with cutaneous leishmaniasis are reported to have a negative impact on quality of life (QOL) and subjected to anxiety, depression due to psychological impairment.

Treatments of dermatological disorders are mainly directed towards the sign and symptoms. Beside these traditional measures of treatment outcome, the assessment of QOL can advance the knowledge to understand the real needs of patients with regard to their disease. It also progresses our knowledge concerning psychological problem attached to the skin disorder which in turn, will be helpful for considering an appropriate therapeutic decision for them. Despite the high endemicity of PKDL in Indian subcontinent, the impact of PKDL on QOL has not been studied previously. Therefore, we aimed to estimate the effect of PKDL on quality of life in Indian PKDL patients by using Dermatology life quality index (DLQI), Short form 36 questionnaire (SF36) and also tried to evaluate the socio-demographic and characteristics of lesions associated with the impairment of QOL.

## Methods

### Study setting and study design

This was a single center, cross-sectional study conducted between February 2016 to June 2016 at Rajendra Memorial Research Institute of Medical sciences (RMRIMS), Indian council of medical Research (ICMR), Patna, Bihar, a permanent institute under administrative control of Govt. of India. We performed this study in a hospital setting, which provides free outpatient and inpatient care. The PKDL patients referred to the hospital supervised by the Department of Clinical Medicine were enrolled by the convenience sampling method.

### Study participants

A total of 92 PKDL patients and 96 comparison participants aged between 16 and 67 years were included in the study. The clinical diagnosis of PKDL was done by rK39 test and by demonstration of leishmanial amastigote through a slit skin smear. Severity of PKDL was graded as mild (very few lesions, usually on the face), moderate (lesions easily visible and generalized) and severe (dense coverage with lesions and little normal skin remains) [8]. All the patients diagnosed with PKDL were admitted in the indoor ward of RMRIMS. A comparison group of healthy people was recruited from the accompanied persons attending hospital along with patients. Comparison group was apparently healthy and free from any disease by physical examination. They were matched with the study group for age, sex, educational level, residential locations, occupation and marital status.

The inclusion criteria were male and female, aged ≥16 years. Patients having skin lesions other than PKDL and having any disability or chronic illness which would have an impact on QOL were excluded. We excluded children, since SF-36 was meant for measuring health related quality of life (HRQOL) among adults only.

### Sample size

No formal sample size calculation was performed. However, all PKDL patients admitted in RMRIMS indoor ward during the study period were the study subjects, provided they were mentally competent and willing to participate. Thus, a total of 186 (92 patients and 96 controls) subjects enrolled in the study. The sample size covered under this study, assumed to be sufficient enough to provide a lead towards the quality of life of PKDL patients.

### Data collection

Data were collected by three questionnaires, one questionnaire that included demographic data.

and clinical features of the lesions such as type, location, duration, severity of the lesions, previous treatment if any. The second questionnaire was the Dermatology Life Quality Index (DLQI) that measured the skin specific quality of life and third one was SF 36 for evaluation of general health status.

#### DLQI

DLQI is a self administered questionnaire designed by Finlay and Khan in 1992, to measure the overall impact of skin diseases on quality of life in patients. It consists of 10 questions categorized into six items, e.g. symptoms and feelings (question 1-2), daily activities (question 3-4), leisure (question 5-6), work and schooling (question 7), personal relationships (question 8-9) and treatment (question 10) during last 7 days [10]. Each question carries four possible responses scored ranged from 0 to 3, giving total DLQI scores of 0-30. The higher score represents a greater impairment of quality of life. The score between 0 and 1 indicates no effect on patient’s quality of life, 2-5 represents small effect, 6-10 moderate effect, 11-20 very large effect, 21-30 indicates the extremely large effect on patients’ quality of life. A validated Hindi version of DLQI questionnaire was used to assess the QOL [11].

#### SF36

The SF36 is self administered questionnaire for evaluation of general health status, comprising of 36 questions grouped into 8 domains, physical functioning, social functioning, role limitations related to physical problems, role limitations related to emotional problems, mental health, vitality, bodily pain and general health perception [12]. A score ranging from 0 (worst measured health) to 100 (best measured health) was assigned for each domain. A higher score represents better health. SF36 was translated into local language.

The aim of the study was explained to the participants. Patients were called individually to fill the questionnaire. A trained investigator was present during filling and all the queries raised by the participants were resolved.

### Data analysis

Data were analyzed by using SPSS software (version 16). Student’s t-test, analysis of variance (ANOVA) was applied for comparison of means and results are expressed as mean ± SD. *P* value less than 0.05 was considered statistically significant.

Pearson correlation between the quantitative variables and DLQI scores were performed. Multivariate linear regression analysis was conducted with DLQI scores as dependent or outcome variable and age, sex, severity and treatment type as independent variables in order to identify their relevance for quality of life of the studied patients.

### Ethics statements

This study was approved by the Institutional Ethical Committee of Rajendra Memorial Research Institute of Medical Sciences (RMIMS), Patna. Informed consent was available in Hindi language. Written Informed consent was taken from all the participants prior to administration of questionnaires. They were also ensured about the anonymity and confidentiality of data.

## Results

In total, 92 patients with PKDL and 96 healthy participants were recruited in the study. The study group consisted of 50 (56.5%) males and 42 (45.6%) females, aged 16-60 years (mean 27.4) while, comparison group (healthy participants) consisted of 53 (55.2%) male and 43 (44.79%) female, aged 18-67 years (mean 29.5).

The mean (± SD) DLQI score was 11.41 ± 4.89 for PKDL cases, indicating a very large impact on quality of life. The highest impact was found for the symptoms and feelings domains; the lowest impact was observed for the personal relationship domain of the DLQI. The mean DLQI scores of different domains are presented in (Table 1).

**Table 1:** DLQI scores in patients with PKDL

DLQI domains	Mean ± SD	Minimum	Maximum
Symptoms and feelings	2.18 ± 1.08	0	6
Daily activities	1.84 ± 1.4	0	6
Leisure	1.86 ± 1.54	0	6
Work and school	1.76 ± 1.26	0	3
Personal relationships	1.68 ± 1.21	0	6
Treatment	2.06 ± 1.05	0	3
Total scores	11.41 ± 4.89	0	30

**Figure 1:**
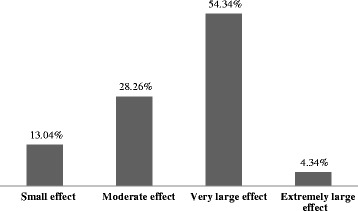
Fig. 1 Impact of PKDL on quality of life in patients

Figure 1 demonstrates the impact of PKDL on quality of life in patients. The maximum number of patients, (54.34%) experienced very large effect and 4.34% of patients had extremely large effect on QOL. The mean scores of DLQI in male and female were 11.28 (± 4.71) and 11.57 (± 5.91) respectively, however, this difference was not statistically significant (*p* = 0.77). The mean scores of the DLQI varies significantly in different age groups (*p* = 0.03). The highest impairment of QOL was found among the patient below 20 years of age. We did not find any significant association in QOL with respect to the marital status (*p* = 0.50) and type of lesions (*p* = 0.07) (Table 2). Patients with nodular lesions had lower QOL when compared to macular or papular lesions. Patients who had lesions for longer duration and present in both exposed and unexposed parts had reported higher DLQI. However, statistically no significant difference was observed with respect to duration (*p* = 0.15) and location (*p* = 0.91) of lesions. The significant effect in QOL was found in the severity of PKDL (*p* = 0.001) and treatment status (*p* = 0.04) (Table 2). Patients with mild lesions had better QOL than moderate and severe lesions. Patients before initiation of treatment had a higher effect on QOL when compared to patients receiving treatment.

**Table 2:** Demographic and Clinical variables associated with Dermatology Life Quality Index scores

	DLQI scores		
Variables	N (%)	Mean ± SD	*P* value
Gender			
Male	50 (54.3)	11.28 ± 4.71	0.77
Female	42 (45.6)	11.57 ± 5.14	
Age (year)			
< 20	31 (33.7)	12.93 ± 4.88	0.03
20-40	48 (52.2)	10.16 ± 4.70	
> 40	13 (14.1)	12.38 ± 4.68	
Marital Status			
Single	49 (53.2)	11.73 ± 4.94	0.5
Married	43 (46.7)	11.04 ± 4.85	
Education			
NFE	23 (25)	12.3 ± 5.12	0.55
Primary School	34 (37)	11.2 ± 4.48	
Secondary School	23 (25)	11.65 ± 5.44	
College	12 (13)	9.83 ± 4.6	
Lesion type			
Macular	31 (33.7)	11.9 ± 4.26	0.07
Papular	14 (15.2)	7.92 ± 4.84	
Nodular	17 (18.5)	12.29 ± 4.28	
Maculopapular	17 (18.5)	12.11 ± 5.85	
Papulonodular	13 (14.1)	11.9 ± 4.85	
Treatment type			0.04
Under treatment	41 (44.5)	10.29 ± 4.82	
Before treatment initiation	51 (55.4)	12.31 ± 4.80	
Severity			
Mild	30 (32.6)	9.16 ± 4.36	0.001
Moderate	42 (45.6)	11.69 ± 4.17	
Severe	20 (21.7)	14.2 ± 5.6	
Duration of disease (year)			
< 1	24 (26.1)	10.1 ± 4.01	0.15
1-5	55 (59.8)	11.5 ± 5.07	
> 5	13 (14.1)	13.3 ± 5.09	
Lesion location			
Exposed part	28 (30.4)	11.28 ± 5.0	0.91
Unexposed part	9 (9.8)	10.88 ± 4.9	
Both	55 (59.8)	11.56 ± 4.9	

*SD* standard deviation, *NFE* no formal education

There is statistically no significant relationship found between total DLQI scores and age.

(*r* = 0.003, *p* = 0.974) as well as duration of disease (*r* = 0.166, *p* = 0.115) (Table 3). Linear regression model of factors affecting the quality of life showed that severity was the only significant predictor of impaired quality of life (*p* < 0.001) and R^2^ = 0.14 indicates that severity accounts for 14% of variation in the overall scores of DLQI (Table 4).

**Table 3:** Correlation analysis between age, disease duration with Dermatology Life Quality Index scores

		Patient age	Disease duration
Total DLQI	r	0.003	0.166
	P	0.974	0.115

*r* correlation coefficient

**Table 4:** Linear regression model of factors affecting Dermatology Life Quality Index

	Beta coefficient	SE	t	*P* value
Age	0.003	4.91	0.033	0.974
Sex	0.030	4.91	0.283	0.778
Severity	0.377	4.55	3.86	<0.001
Treatment Type	0.207	4.81	2.00	0.048

*SE* standard error

The mean individual scores of SF36 for the study and comparison group are shown in (Table 5). Apart from body pain, the study group had lower scores than the comparison group. The PKDL cases showed significant difference in various aspects of QOL such as mental health (MH), social functioning (SF), bodily pain (BP) and general health (GH) as compared to control group. There was no significant difference (*P* > 0.05) between two groups with respect to the physical functioning (PF), role physical (RP), role emotional (RE) and vitality (VT).

**Table 5:** SF36 scores of the study and control groups

SF36 Domains	PKDL Group (*n* = 92) Mean ± SD	Control Group (*n* = 96) Mean ± SD	*P* value
Physical Functioning (PF)	93.82 ± 12.73	95.05 ± 11.36	0.44
Role Physical (RP)	59.11 ± 31.64	60.41 ± 31.13	0.80
Role Emotional (RE)	43.06 ± 10.54	60.56 ± 34.21	0.14
Vitality (VT)	53.16 ± 2.27	56.73 ± 2.09	0.31
Mental Health (MH)	55.03 ± 20.15	61.53 ± 20.89	0.02
Social Function (SF)	45.63 ± 29.82	70.07 ± 27.51	0.00
Body Pain (BP)	83.32 ± 3.35	72.65 ± 3.59	0.04
General Health (GH)	41.92 ± 2.05	51.33 ± 24.31	0.00

*SD* standard deviation

## Discussion

There are studies on QOL for other skin diseases, however to the best of our knowledge there was no study on QOL in patients with PKDL. A study in cutaneous leishmaniasis (CL) revealed a negative impact of the disease on QOL and concluded that CL has a moderate to large negative effect on the QOL [13]. Another study by Yanik et al. reported that CL not only impairs the QOL but also the psychological functioning in the form of depression and anxiety in contrast to control group [14]. Study on QOL in patients with PKDL may be helpful in identifying those cases whose QOL is highly affected**.** To provide a more comprehensive assessment of the burden associated with PKDL, dermatology specific (DLQI) and general health specific (SF36) questionnaires were used together in the present study.

We evaluated a high impact of PKDL on patients QOL. The average DLQI scores 11.41, indicating a very large impairment of QOL. The highest score (2.18) was obtained in symptoms and feelings domain which represents how itching or painful were the lesions, embarrassment owned by the patients due to PKDL lesions. This finding was concordant with the result published in other skin related disorder such as psoriasis [15], contact dermatitis [16], primary cutaneous amyloidosis [17]. Treatment also showed equally comparable DLQI score (2.06). The recommended treatment options are miltefosine at 2.5 mg/kg for 12 weeks and amphotericin B in the dose of 1 mg/kg in 5% dextrose IV, alternate days for 20 injections in 3 to 4 courses at 15 days interval. These treatments are lengthy and also associated with numerous side effects. The possible explanation for such high DLQI score may be the longer hospitalization and prolonged treatment. The lowest score was found for the personal relationship domain of DLQI, which was in congruence to the findings of a study conducted on cutaneous lesihmaniasis in Brazil [13].

Diagnosis and treatment of PKDL is of outmost public health importance in Indian sub-continent. It is vital for the VL elimination program, as PKDL is considered to be a reservoir of infection and may lead to increase in VL incidence rapidly. Moreover, effective, safe and shorter duration of PKDL treatment is essentially important, as it leads to the normal skin appearance thereby leading to prevention of stigmatization and thus improvement of QOL in the affected patient. Apart from the treatment by qualified medical personnel, the service of a psychologist may be needed for up gradation of QOL in an affected PKDL patient**.**

In our study, the mean DLQI scores of PKDL patients were 11.41, showing better result when compared to the other chronic skin disorders such as burns (17.7) [18], psoriasis (12.8) [19] and epidermolysis bullosa (12.1) [20] but worse than cutaneous leishmaniasis (5.87) [21], acne vulgaris (8.18) [22], alopecia (8.3) [23] and primary cutaneous amyloidosis (9.05) [17]. Around half of the patients (54.34%) in our study reported to have very large effect on QOL, whereas in cutaneous leishmaniasis only 15% patients experienced the same [21].

DLQI score revealed that quality of life (QOL) of PKDL patients did not vary significantly with respect to gender, marital status and type of skin lesions, except for nodular lesions which had poor QOL. The possible explanation may be the fact that nodular lesions generally appears on the exposed part of the body; more markedly on face, which lead to clinical and social discomfort. Nodular lesions contained a large amount of leishmania parasites, which may possibly play a significant role in transmission of the disease and thus have public health importance.

We did not find any significant difference for gender, similar observation was reported by Behrooze et al. in Iranian cutaneous leishmaniasis patients [21]. However, it is a common perception that females are more likely to have lower QOL than males as they are more concerned about their beauty and social relationship. The mean score of DLQI for age found under this study revealed that late adolescents had a higher degree of impairment of QOL than the adult. As the late adolescents are more conscious about their self esteem and self appearance, skin lesions may put an effect on their mental health and social obligations. Patients with no formal education were found to have lower quality of life. This may be explained as patients without formal education might be less aware of PKDL and/or pay less attention to the skin lesions at the initial stage, which appears as a small macule and later on gradually convert to more severe form thereby, leading to poor QOL. Patients with severe PKDL and persistence of lesions for several years were reported to have lower quality of life. Lesions of PKDL initially appear as hypopigmented macules but progressively converted to papules, nodules or mixture of these. Moreover, with increasing duration plaques and ulceration may develop. This ultimately affects their aesthetic appearance, physical and mental well-being. Therefore, patient’s education and awareness could be a key strategy to prevent severity of lesions and treatment delay.

For the first time, we report the impact of PKDL on QOL measured by SF-36, using comparison groups. The PKDL cases showed significant difference in various aspects of QOL viz. mental health (MH), social functioning (SF), bodily pain (BP) and general health (GH) as compared to control group. This finding is similar to a study conducted by Sheng et al. on Primary Cutaneous Amyloidosis [17]. PKDL patients also achieved lower scores as compared to control for role emotional, mental health and social functioning domain of SF36 test. This probably indicated that the psychological and social factors interfere with the QOL of PKDL patients. How these psychosocial parameters impair the QOL is needed to be explored in greater detail.

### Limitations

Socioeconomic status is associated with education and employment which in turn could have modulating association with disease severity. In this study, QOL scoring was poor, although statistically insignificant, among those with no/lower formal education. Employment/financial status of patients’ was not assessed in this study. These factors are known to have association with poor QOL [24]. Therefore, the findings of this study should be interpreted accordingly. The other limitation of this study was small sample size, which may hamper comparison among the different subgroups and may limit the applicability of results.

## Conclusions

The present study has shown that the quality of life is highly impaired in patients with PKDL especially symptoms, feelings and treatment domain. Hence, a dermatologist should consider the psychological aspects of the disease in conjugation with therapeutic intervention to formulate an effective patient care management plan for PKDL. Issue related to QOL for PKDL patients need further research using clinical and epidemiological reviews to better understand the natural history, pathogenesis and long term impact of persisting manifestation following nodular/maculopapular/papulonodular lesions for PKDL. This has important implications for developing intervention programs in the country with high risk of PKDL increase. Studies with larger number of patients are recommended to evaluate the effects of location, number, size and duration of PKDL lesions, as well as the effects of treatment and residual scars on the QOL of patients. The possibility of using the DLQI as an outcome measure in future clinical studies on PKDL should also need to be kept in mind.
